# Estimation of variance components and prediction of breeding values based on group records from varying group sizes

**DOI:** 10.1186/s12711-018-0413-y

**Published:** 2018-08-14

**Authors:** Guosheng Su, Per Madsen, Bjarne Nielsen, Tage Ostersen, Mahmoud Shirali, Just Jensen, Ole F. Christensen

**Affiliations:** 1 Center for Quantitative Genetics and Genomics, Department of Molecular Biology and Genetics, Aarhus University, 8830 Tjele, Denmark; 2 SEGES, Pig Research Centre, 1609 Copenhagen, Denmark

## Abstract

**Background:**

Records on groups of individuals rather than on single individuals could be valuable for predicting breeding values (BV) of the traits that are difficult or costly to measure individually, such as feed intake in pigs or beef cattle. Here, we present a model, which handles group records from varying group sizes and involves multiple fixed and random effects, for estimating variance components and predicting BV. Moreover, using simulation, we investigated the efficiency of group records for predicting BV in situations with various group sizes and structures, and factors that affect the trait.

**Results:**

The results show that the presented model for group records worked well and that variances estimated from group records with varying group sizes were consistent with those estimated from individual records, but with larger standard errors. Ignoring litter and pen effects had very little or no influence on the accuracy of estimated BV (EBV) obtained from group records. However, ignoring litter effects resulted in biased estimates of additive genetic variance and EBV. The presence of litter and pen effects on phenotypes decreased the accuracy of EBV although the prediction model fitted both effects. Having more littermates in the same pen led to a higher accuracy of EBV. The decay of EBV accuracy with increasing group size was more marked for scenarios with litter and pen effects than without. When litters of six individuals were divided into two pens, accuracies of EBV obtained from group records with a size up to 12 (average 9.6) and up to 24 (average 19.2) were 66.6 and 57.6% of those estimated from individual records in the scenario with litter and pen effects on phenotypes. These percentages reached 77.0 and 68.4% in the scenario without litter and pen effects on phenotypes.

**Conclusions:**

Our results indicate that the model works appropriately for the analysis of group records from varying group sizes. Using group records for genetic evaluation of traits such as feed intake in pig is feasible and the efficiency of the resulting estimates depends on the size and structure of the groups and on the magnitude of the variances for litter and pen effects.

## Background

The amount of available data is a key factor that affects accuracy of selection in a breeding program. For some traits, individual records are difficult or expensive to measure, making it difficult to obtain a sufficient amount of data. An example is feed intake, which is one of the most important traits in livestock production, since feed represents 60 to 70% of the total cost in modern commercial livestock production systems [1–3]. Feed intake at the individual level is usually measured by electronic feeders that can recognize each individual in the pen/barn (pig/cattle), or by isolating an animal in a cage (poultry). Because individual recording of feed intake is expensive, individual feed intake is usually only measured for a small proportion of selection candidates. For most selection candidates, their BV are predicted using information from their relatives that have individual records of feed intake and information on correlated traits such as growth or production traits that are usually recorded individually. The lack of phenotypic data on feed intake is a barrier in the genetic progress of feed efficiency. Therefore, alternatives to measuring individual feed intake are required in order to improve the accuracy of genetic evaluation of feed efficiency.

One alternative is to use data of feed intake at the group level. In pig farms, a group of pigs (e.g., 12–15 pigs) are usually kept in a pen during a particular growth period (e.g., from 30 to 100 kg). Although it is costly to measure feed intake individually due to the expensive equipment required, total feed intake at the pen level is relatively easy to measure by manually or automatically weighing the feed that is provided in the feeder during the trial and the remaining feed at the end of the trial. Many studies have reported the estimation of variance components [4–6] and prediction of BV using group records [5–8]. However, in all these studies only additive genetic and residual effects were considered as random effects in the model, and they analyzed data of equal group sizes or set a constant group size to model the residual variance when analyzing data of varying group sizes [8]. Thus, it is necessary to extend these approaches to handle multiple fixed and random effects, such as litter and pen effects on feed intake in pigs, and investigate the efficiency of using group records for predicting BV in different scenarios.

The objectives of this study were to (1) model group records from varying group sizes, considering both additive genetic and non-genetic random effects (litter and pen effects); (2) interpret variance components at the group level; (3) assess the variances estimated from group records; and (4) compare prediction accuracy based on group and individual records in different scenarios.

## Methods

In this section, first we describe the model used to fit group records with unequal group sizes; second, we illustrate variances at the group level and variance components estimated from group records; third, we present the simulation of data for various scenarios by mimicking feed intake in pigs; and finally, we describe the analysis of data to compare the efficiency of using group versus individual records for predicting BV in different scenarios and with different models.

### Model and variance components for group records

Assume that phenotypes for a trait (vector of individual records ${\mathbf{y}}$), such as feed intake in pigs, are affected by additive genetic effects (${\mathbf{a}}$), litter effects (${\mathbf{l}}$), pen effects (${\mathbf{c}}$), and some fixed effects (${\mathbf{b}}$), along with residual effects (${\mathbf{e}}$), resulting in the following linear mixed model for individual records:

1 $${\mathbf{y}} = {\mathbf{Xb}} + {\mathbf{Z}}_{{\mathbf{l}}} {\mathbf{l}} + {\mathbf{Z}}_{{\mathbf{c}}} {\mathbf{c}} + {\mathbf{Z}}_{{\mathbf{a}}} {\mathbf{a}} + {\mathbf{e}},$$

where ${\mathbf{X}}$, ${\mathbf{Z}}_{{\mathbf{l}}}$, ${\mathbf{Z}}_{{\mathbf{c}}}$, and ${\mathbf{Z}}_{{\mathbf{a}}}$ are incidence matrices linking ${\mathbf{b}}$, ${\mathbf{l}}$, ${\mathbf{c}}$ and ${\mathbf{a}}$ to ${\mathbf{y}}$. Litter effects reflect the continuous effects due to common environment before weaning, while pen effects represent common environment effects during the grow-finish period. It is assumed that the random effects have the following distributions:

$${\mathbf{l}}\sim \it{N}\left( {\bf{0}, {\mathbf{I}}\sigma_{{\mathbf{l}}}^{2} } \right),\quad {\mathbf{c}}\sim N\left( {\bf{0}, {\mathbf{I}}\sigma_{{\mathbf{c}}}^{2} } \right),\quad {\mathbf{a}}\sim N\left( {\bf{0}, {\mathbf{A}}\sigma_{{\mathbf{a}}}^{2} } \right),{\textrm{ and}}\quad {\mathbf{e}}\sim N\left( {\bf{0}, {\mathbf{I}}\sigma_{{\mathbf{e}}}^{2} } \right),$$

where ${\mathbf{I}}$ is an identity matrix, ${\mathbf{A}}$ is the additive genetic relationship matrix, $\sigma_{{\mathbf{l}}}^{2}$, $\sigma_{{\mathbf{c}}}^{2}$, and $\sigma_{{\mathbf{a}}}^{2}$ are the variance of litter effects (hereafter referred to as litter variance), the variance of pen effects (hereafter referred to as pen variance), and additive genetic variance, respectively. The expectation and variance of ${\mathbf{y}}$ are:

$${\text{E}}\left( {\mathbf{y}} \right) = {\mathbf{Xb}},$$

and $${\text{V}}\left( {\mathbf{y}} \right) = {\mathbf{Z}}_{{\mathbf{l}}} {\mathbf{Z}}_{{\mathbf{l}}}^{{\prime }} \sigma_{{\mathbf{l}}}^{2} + {\mathbf{Z}}_{{\mathbf{c}}} {\mathbf{Z}}_{{\mathbf{c}}}^{{\prime }} \sigma_{{\mathbf{c}}}^{2} + {\mathbf{Z}}_{{\mathbf{a}}} {\mathbf{AZ}}_{{\mathbf{a}}}^{{\prime }} \sigma_{{\mathbf{a}}}^{2} + {\mathbf{I}}\sigma_{{\mathbf{e}}}^{2}$$.

For a trait measured at the group level, for example, aggregated feed intake in a pen, the model for group records (here a pen is considered as a group) corresponding to the model for individual records can be written as:

2 $${\mathbf{Ty}} = {\mathbf{T}}\left( {{\mathbf{Xb}} + {\mathbf{Z}}_{{\mathbf{l}}} {\mathbf{l}} + {\mathbf{Z}}_{{\mathbf{c}}} {\mathbf{c}} + {\mathbf{Z}}_{{\mathbf{a}}} {\mathbf{a}} + {\mathbf{e}}} \right),$$

where ${\mathbf{T}}$ is an indicator matrix that links individual records to groups with number of rows equal to the number of groups and number of columns equal to the total number of animals with records. Element (${\mathbf{T}}_{ij}$) of ${\mathbf{T}}$ is 1 if the $j$th animal belongs to the $i$th group, and otherwise 0. Matrix ${\mathbf{T}}$ also sums variables for each level of particular factor within a group.

The model for group records can be written in the typical form of a linear mixed model:

3 $${\mathbf{y}}^{*} = {\mathbf{X}}^{*} {\mathbf{b}} + {\mathbf{Z}}_{{\mathbf{l}}}^{*} {\mathbf{l}} + {\mathbf{Z}}_{{\mathbf{c}}}^{*} {\mathbf{c}} + {\mathbf{Z}}_{{\mathbf{a}}}^{*} {\mathbf{a}} + {\mathbf{e}}^{*} ,$$

where $${\mathbf{y}}^{*} = {\mathbf{Ty}}$$, $${\mathbf{X}}^{*} = {\mathbf{TX}}$$, $${\mathbf{Z}}_{{\mathbf{l}}}^{*} = {\mathbf{TZ}}_{{\mathbf{l}}}$$, $${\mathbf{Z}}_{{\mathbf{c}}}^{*} = {\mathbf{TZ}}_{{\mathbf{c}}}$$, $${\mathbf{Z}}_{{\mathbf{a}}}^{*} = {\mathbf{TZ}}_{{\mathbf{a}}}$$, and $${\mathbf{e}}^{*} = {\mathbf{Te}}$$.

Thus, ${\mathbf{y}}^{*}$ is the vector of group records with element $y_{i}^{*} = {\mathbf{t}}_{i} {\mathbf{y}} = \mathop \sum \nolimits_{j = 1}^{{n_{gi} }} y_{ij}$, where ${\mathbf{t}}_{i}$ is the $i$th row of matrix ${\mathbf{T}},$ with elements indicating the animals in the $i$th group, and $n_{gi}$ is the number of animals in the $i$th group. For matrices ${\mathbf{X}}^{*}$, ${\mathbf{Z}}_{{\mathbf{l}}}^{*}$, ${\mathbf{Z}}_{{\mathbf{c}}}^{*}$ and ${\mathbf{Z}}_{{\mathbf{a}}}^{*}$, the number of rows is equal to the number of groups and the number of columns is equal to that of matrices ${\mathbf{X}}$, ${\mathbf{Z}}_{{\mathbf{l}}}$, ${\mathbf{Z}}_{{\mathbf{c}}}$, and ${\mathbf{Z}}_{{\mathbf{a}}}$, respectively. Element ${\mathbf{X}}_{{\left( {ij} \right)}}^{*}$ is either the number of animals in the $i$th group at the $j$th level of the fixed effect, or the sum of elements in the $i$th group for a regression covariable. Element ${\mathbf{Z}}_{{{\mathbf{l}}\left( {ij} \right)}}^{*}$ is the number of animals of the $j$th litter included in the $i$th group, and the number of non-zero elements in the $i$th row of ${\mathbf{Z}}_{{\mathbf{l}}}^{*}$ is equal to the number of litters involved in the $i$th group. Element ${\mathbf{Z}}_{{{\mathbf{c}}\left( {ij} \right)}}^{*}$ is the number of animals of the $j$th pen included in the $i$th group. When considering a pen as a group, ${\mathbf{Z}}_{{\mathbf{c}}}^{*}$ is a diagonal matrix with diagonal element ${\mathbf{Z}}_{{{\mathbf{c}}\left( {ii} \right)}}^{*}$ equal to group size. Element ${\mathbf{Z}}_{{{\mathbf{a}}\left( {ij} \right)}}^{*}$ is 1 or 0 to indicate if the $j$th animal is in the $i$th group. Thus, the number of 1 s in the $i$th row of ${\mathbf{Z}}_{{\mathbf{a}}}^{*}$ is equal to the number of animals in the $i$th group. The expectation and variance of ${\mathbf{y}}^{*}$ are:

$${\text{E}}({\mathbf{y}}^{*} ) = {\mathbf{X}}^{\varvec{*}} {\mathbf{b}}\quad{\text{and}}\;{\text{V}}\left( {{\mathbf{y}}^{*} } \right) = {\mathbf{Z}}_{{\mathbf{l}}}^{*} {\mathbf{Z}}_{{\mathbf{l}}}^{{*{\prime }}} \sigma_{{\mathbf{l}}}^{2} + {\mathbf{Z}}_{{\mathbf{c}}}^{*} {\mathbf{Z}}_{{\mathbf{c}}}^{{*{\prime }}} \sigma_{{\mathbf{c}}}^{2} + {\mathbf{Z}}_{{\mathbf{a}}}^{*} {\mathbf{AZ}}_{{\mathbf{a}}}^{{*{\prime }}} \sigma_{{\mathbf{a}}}^{2} + {\mathbf{R}}\sigma_{{\mathbf{e}}}^{2} ,$$

where ${\mathbf{R}}$ is a diagonal matrix with ${\mathbf{R}}_{{\left( {ii} \right)}} = n_{gi}$.

Let ${\mathbf{z}}_{{\mathbf{l}}}^{*}$, ${\mathbf{z}}_{{\mathbf{c}}}^{*}$, and ${\mathbf{z}}_{{\mathbf{a}}}^{*}$ denote the rows of ${\mathbf{Z}}_{{\mathbf{l}}}^{*}$, ${\mathbf{Z}}_{{\mathbf{c}}}^{*}$ and ${\mathbf{Z}}_{{\mathbf{a}}}^{*}$, respectively. For a particular group (here a pen = a group) with a size of $n_{g}$, the variance of the cumulative pen effect for the group is:

4 $$\sigma_{{{\mathbf{c}}^{*} }}^{2} = {\mathbf{z}}_{{\mathbf{c}}}^{*} {\mathbf{z}}_{{\mathbf{c}}}^{{*{\prime }}} \sigma_{{\mathbf{c}}}^{2} = n_{{\mathbf{g}}}^{2} \sigma_{{\mathbf{c}}}^{2} .$$

The definition of the pen variance at the group level ($\sigma_{{{\mathbf{c}}^{*} }}^{2}$) is based on the concept that the pen effect is the same for all animals j and j′ within the same pen, i.e. $Cov\left( {c_{j} ,c_{{j^{\prime}}} } \right) = \sigma_{{\mathbf{c}}}^{2}$. Thus,

$$\sigma_{{{\mathbf{c}}^{*} }}^{2} = Var\left( {\mathop \sum \limits_{j = 1}^{{n_{g} }} c_{j} } \right) = n_{g} \sigma_{{\mathbf{c}}}^{2} + n_{g} \left( {n_{g} - 1} \right)Cov\left( {c_{j} ,c_{j'} } \right) = n_{g}^{2} \sigma_{{\mathbf{c}}}^{2} \text{.}$$

In the next section, we show that although one pen (i.e., one group) has only one group record, it is possible and necessary to include pen effect in the model for group records where the number of animals per pen varies.

The variance of the cumulative litter effect in a particular group is:

5 $$\sigma_{{{\mathbf{l}}^{*} }}^{2} = {\mathbf{z}}_{{\mathbf{l}}}^{*} {\mathbf{z}}_{{\mathbf{l}}}^{{*{\prime }}} \sigma_{{\mathbf{l}}}^{2} = \mathop \sum \limits_{k = 1}^{{N_{L} }} n_{lk}^{2} \sigma_{{\mathbf{l}}}^{2} ,$$

where $N_{L}$ is the number of litters within a group and $n_{lk}$ is the number of littermates from litter $k$ in the group. The definition of litter variance at the group level ($\sigma_{{{\mathbf{l}}^{*} }}^{2}$) is based on the concept that if individuals $j$ and $j^{{\prime }}$ are littermates, $Cov\left( {l_{j} ,l_{j'} } \right) = \sigma_{{\mathbf{l}}}^{2}$, or else $Cov\left( {l_{j} ,l_{{j{\prime }}} } \right) = 0$. Let $m$ and $m^{{\prime }}$ denote individuals within litter $k$,

$$\begin{aligned} \sigma_{{{\mathbf{l}}^{ *} }}^{2} & = Var\left( {\mathop \sum \limits_{j = 1}^{{n_{g} }} l_{j} } \right) = \mathop \sum \limits_{k = 1}^{{N_{L} }} Var\left( {\mathop \sum \limits_{m = 1}^{{n_{lk} }} l_{km} } \right) \\ & = \mathop \sum \limits_{k = 1}^{{N_{L} }} \left( {n_{lk} \sigma_{\text{l}}^{2} + n_{lk} \left( {n_{lk} - 1} \right){\text{Cov}}\left( {l_{km} ,l_{km'} } \right)} \right) = \mathop \sum \limits_{k = 1}^{{N_{L} }} n_{lk}^{2} \sigma_{\text{l}}^{2} . \\ \end{aligned}$$

The variance of the cumulative additive genetic effect for a particular group is:

6 $$\sigma_{{{\text{a}}*}}^{2} = {\mathbf{z}}_{{\mathbf{a}}}^{*} {\mathbf{Az}}_{{\mathbf{a}}}^{{*{\prime }}} \sigma_{{\text{a}}}^{2} = \mathop \sum \limits_{{j = 1\left( {j^{{\prime }} = j} \right)}}^{{n_{g} }} {\mathbf{A}}_{{jj^{{\prime }} }}^{*} \sigma_{{\text{a}}}^{2} + \mathop \sum \limits_{j = 1}^{{n_{g} }} \mathop \sum \limits_{{j^{{\prime }} = 1\left( {j^{{\prime }} \ne j} \right)}}^{{n_{g} }} {\mathbf{A}}_{{jj^{{\prime }} }}^{*} \sigma_{{\text{a}}}^{2} ,$$

where ${\mathbf{A}}^{*}$ is a sub-${\mathbf{A}}$ matrix for the animals in that particular group. The definition of the additive genetic variance at the group level ($\sigma_{{{\text{a}}*}}^{2}$) is based on the concept that the additive genetic covariance between individual $j$ and $j^{{\prime }}$ is $Cov\left( {a_{j} ,a_{{j^{\prime}}} } \right) = {\mathbf{A}}_{jj'}^{*} \sigma_{{\text{a}}}^{2}$. Thus,

$$\begin{aligned} \sigma_{{{\text{a}}*}}^{2} & = Var\left( {\mathop \sum \limits_{j = 1}^{{n_{g} }} a_{j} } \right) \\ & = \mathop \sum \limits_{j = 1}^{{n_{g} }} Var(a_{j} ) + \mathop \sum \limits_{j = 1}^{{n_{g} }} \mathop \sum \limits_{{j^{{\prime }} = 1\left( {j^{{\prime }} \ne j} \right)}}^{{n_{g} }} Cov(a_{j} ,a_{{j^{\prime}}} ) \\ & = \mathop \sum \limits_{{j = 1\left( {j^{{\prime }} = j} \right)}}^{{n_{g} }} {\mathbf{A}}_{{jj^{{\prime }} }}^{*} \sigma_{\text{a}}^{2} + \mathop \sum \limits_{j = 1}^{{n_{g} }} \mathop \sum \limits_{{j^{{\prime }} = 1\left( {j^{{\prime }} \ne j} \right)}}^{{n_{g} }} {\mathbf{A}}_{{jj^{{\prime }} }}^{*} \sigma_{{\text{a}}}^{2} . \\ \end{aligned}$$

In other words, $\sigma_{{{\text{a}}*}}^{2}$ is equal to $\sigma_{\text{a}}^{2}$ times the sum of the elements in the sub-${\mathbf{A}}$ matrix for the animals in that particular group.

Assuming that residual effects between individuals *j* and *j*′ are independent, i.e., $Cov\left( {e_{j} ,e_{{j^{{\prime }} }} } \right) = 0$, where $j$ and $j^{{\prime }}$ are not the same animal, the variance of the cumulative residual effect in a group is:

7 $$\sigma_{{{\mathbf{e}}*}}^{2} = Var\left( {\mathop \sum \limits_{j = 1}^{{n_{g} }} e_{j} } \right) = n_{g} \sigma_{{\mathbf{e}}}^{2} .$$

Therefore, as long as the model for group records is consistent with the model for individual records and the model is appropriate, estimates of variance components are consistent from both types of records. Comparison of Eq. (4) with Eq. (7) shows that the pen variance at the group level has a quadratic relationship with the number of pen mates, while residual variance at the group level has a linear relationship with the number of pen mates. This indicates that, in theory, a model for group records should include pen effects because it is difficult to handle the distribution of the residuals when they include pen effects and group sizes vary. It should be noted that ${\mathbf{Z}}_{{\mathbf{c}}}^{*} {\mathbf{Z}}_{{\mathbf{c}}}^{{*{\prime }}}$ is a diagonal matrix with diagonal elements equal to $n_{gi}^{2}$ when considering pen as the group. If group (pen) size is constant, ${\mathbf{Z}}_{{\mathbf{c}}}^{*} {\mathbf{Z}}_{{\mathbf{c}}}^{{*{\prime }}} = n_{g}^{2} {\mathbf{I}}$ and ${\mathbf{R}} = n_{g} {\mathbf{I}}$, thus ${\mathbf{Z}}_{{\mathbf{c}}}^{*} {\mathbf{Z}}_{{\mathbf{c}}}^{{*{\prime }}} = n_{g} {\mathbf{R}}$. In such a case, the covariance matrix for pen effects is proportional to the covariance matrix of residuals, and thus, pen and residual effects cannot be separated. Similarly, if each pen contains one whole litter, ${\mathbf{Z}}_{{\mathbf{l}}}^{*} {\mathbf{Z}}_{{\mathbf{l}}}^{{*{\prime }}} = {\mathbf{Z}}_{{\mathbf{c}}}^{*} {\mathbf{Z}}_{{\mathbf{c}}}^{*'}$, and thus, litter effect and pen effects are not identifiable.

An example to demonstrate the model for group records and variance components is shown in the “Appendix”.

### Simulation of data

Estimation of variance components and prediction of BV using group records were evaluated using simulated data of individual feed intake in a pig population. To simplify the simulation procedure, the simulated populations were under random selection and mating, sows had first parity only, and generations did not overlap. Each generation had 30 sires, each sire was mated to 20 dams, and each litter had six individuals available for testing. In total, eight generations of data were simulated. Only the last five generations of phenotypic data were used in the analysis but pedigree was traced back to the base population.

Two sets of phenotypic values (${\text{y}}_{\text{alp}}$ and ${\text{y}}_{\text{a}}$) were simulated. In phenotype 1, ${\text{y}}_{\text{alp}}$ = litter effect + pen effect + additive genetic effect + residual, and the variance components were $\sigma_{{\mathbf{a}}}^{2}$ = 120, $\sigma_{{\mathbf{l}}}^{2}$ = 40, $\sigma_{{\mathbf{c}}}^{2}$ = 40, and $\sigma_{{\mathbf{e}}}^{2}$ = 200. In phenotype 2, ${\text{y}}_{\text{a}}$ = additive genetic effect + residual, and the variance components were $\sigma_{{\mathbf{a}}}^{2}$ = 120, and $\sigma_{{\mathbf{e}}}^{2}$ = 280. It was assumed that ${\mathbf{l}}\sim N\left( {\bf{0}, {\mathbf{I}}\sigma_{{\mathbf{l}}}^{2} } \right)$, ${\mathbf{c}}\sim N\left( {\bf{0}, {\mathbf{I}}\sigma_{{\mathbf{c}}}^{2} } \right)$, ${\mathbf{a}}\sim N\left( {\bf{0}, {\mathbf{A}}\sigma_{{\mathbf{a}}}^{2} } \right)$, and ${\mathbf{e}}\sim N\left( {\bf{0}, {\mathbf{I}}\sigma_{{\mathbf{e}}}^{2} } \right)$.

A group record was defined as the sum of individual records within a pen. To investigate the effect of different group structures on estimates of variance components and BV, three group structures with an initial pen/group size of 12 were generated, i.e., (1) L_1×6_: all individuals of a litter were assigned to the same pen and thus, a pen included two litters; (2) L_2×3_: a litter was randomly divided into two sub-litters of size 3, which were randomly allocated to pens, and thus a pen included pigs from four litters; and (3) L_ran_: individuals of a litter were randomly distributed to pens. Scenario L_2×3_ was taken as the base scenario for detailed analysis. To investigate the effect of group size on genetic evaluation, different group sizes were simulated, ranging from 3 to 30. In this setting, animals were allocated to pens in two ways, i.e. (1) sub-litters (each with three individuals) were randomly allocated to pens, and (2) individuals randomly allocated to pens. The construction of groups was performed within generations.

In practice, group sizes in a dataset are not constant due to differences in pen size, rearing density between farms, etc. To create various group sizes within a scenario, 20% of the individuals were randomly deleted (but all breeding animals were kept). Thus, group sizes within a scenario followed a binomial distribution with a mean group size equal to 80% of the pre-designed size.

For each scenario, 100 replicates were generated and analyzed, and the mean and standard deviation of these 100 replicates are presented. The standard deviation reflects standard error for an estimate of a parameter.

### Statistical models to analyze the simulated data

For both datasets with or without simulated litter ($l$) and pen (*c*) effects, variance components were estimated and breeding values were predicted using the following four models:

$$\begin{aligned} {\mathbf{y}}^{*} & = 1\mu + {\mathbf{Z}}_{{\mathbf{l}}}^{*} {\mathbf{l}} + {\mathbf{Z}}_{{\mathbf{c}}}^{*} {\mathbf{c}} + {\mathbf{Z}}_{{\mathbf{a}}}^{*} {\mathbf{a}} + {\mathbf{e}}^{*} ,&\quad ({\text{M}}_{\text{ALP}} ) \\ {\mathbf{y}}^{*} & = 1\mu + {\mathbf{Z}}_{{\mathbf{l}}}^{*} {\mathbf{l}} + {\mathbf{Z}}_{{\mathbf{a}}}^{*} {\mathbf{a}} + {\mathbf{e}}_{{\mathbf{c}}}^{*} ,&\quad ({\text{M}}_{\text{AL}} ) \\ {\mathbf{y}}^{*} & = 1\mu + {\mathbf{Z}}_{{\mathbf{c}}}^{*} {\mathbf{c}} + {\mathbf{Z}}_{{\mathbf{a}}}^{*} {\mathbf{a}} + {\mathbf{e}}_{{\mathbf{l}}}^{*} ,&\quad ({\text{M}}_{\text{AP}} ) \\ {\mathbf{y}}^{*} & = 1\mu + {\mathbf{Z}}_{{\mathbf{a}}}^{*} {\mathbf{a}} + {\mathbf{e}}_{{{\mathbf{cl}}}}^{*}. &\quad ({\text{M}}_{\text{A}} ) \\ \end{aligned}$$

The datasets of individual records were also analyzed using the models for individual records (M_ALP_i_, M_AL_i_, M_AP_i_ and M_A_i_, corresponding to M_ALP_, M_AL_, M_AP_ and M_A_). In the analysis of the effect of different group sizes and structures on prediction of BV, M_ALP_ with true (simulated) variances was used to predict BV from phenotypes with litter and pen effects, and M_A_ with true variances was used to predict BV from phenotypes without litter and pen effects.

Variance components were estimated by the average information (AI) REML procedure [9] and BV were predicted with the BLUP method [10]. All analyses were performed with the DMU package [11]. The efficiency of using group versus individual records for predicting BV was assessed by accuracy and bias of BV prediction. Accuracy was calculated as the correlation between predicted and true BV for all animals with phenotype data. Bias was evaluated by regression of true on predicted BV.

## Results

### Estimates of variance components from group models

Variance components estimated from group or individual records of the basic scenario (L_2×3_) using different models are in Table 1. Based on individual records for which phenotypes included litter and pen effects, as expected, variance components were estimated without bias using the full model (M_ALP_). On the one hand, the models that ignored litter effect (M_AP_ and M_A_) greatly overestimated the additive genetic variance, which indicates that most of the litter effect moved to the additive genetic effect due to the strong genetic relationship between littermates (r = 0.5). Model M_AP_ also overestimated pen variance, which suggests that part of the litter effect moved to the pen effect. On the other hand, the model that ignored pen effects (M_AL_) led to an unbiased estimate of additive genetic variance, but overestimated litter variance and increased residual variance, indicating that one part of the pen effect moved to the litter effect and the other part moved to the residual effect.

**Table 1:** Estimates of variance components [mean (SD) over 100 replicates] from group or individual records when using different models for the base scenario (L_2×3_)

Litter and pen effect	Record	Model	Additive genetic variance $\left( {\sigma_{\text{a}}^{2} } \right)$	Litter variance $(\sigma_{\text{l}}^{2} )$	Pen variance $(\sigma_{\text{c}}^{2} )$	Residual variance $(\sigma_{\text{e}}^{2} )$
Yes		Simulated	120	40	40	200
	Group	M_A_	133.1 (28.8)			661.9 (35.7)
		M_AL_	118.8 (26.5)	48.7 (22.6)		558.0 (58.1)
		M_AP_	129.7 (27.5)		46.2 (16.9)	226.1 (155.6)
		M_ALP_	118.5 (25.7)	38.8 (22.4)	40.4 (17.0)	209.3 (150.5)
	Individual	M_A_	175.3 (10.1)			236.9 (6.2)
		M_AL_	119.5 (11.1)	55.9 (3.9)		223.5 (6.8)
		M_AP_	158.1 (9.3)		46.1 (2.6)	204.1 (5.8)
		M_ALP_	119.4 (10.1)	39.9 (3.3)	40.0 (2.4)	199.6 (6.3)
No		Simulated	120	0	0	280
	Group	M_A_	118.0 (17.6)			281.9 (20.3)
		M_AL_	114.6 (18.0)	5.4 (7.2)		271.2 (23.2)
		M_AP_	116.5 (17.7)		4.0 (6.5)	244.7 (59.7)
		M_ALP_	114.5 (18.9)	4.9 (7.0)	3.6 (6.1)	238.2 (57.8)
	Individual	M_A_	119.2 (9.1)			279.4 (6.5)
		M_AL_	118.0 (9.0)	1.0 (1.5)		279.2 (6.5)
		M_AP_	119.0 (9.0)		0.5 (0.7)	279.0 (6.5)
		M_ALP_	118.0 (8.9)	0.9 (1.4)	0.5 (0.7)	278.8 (6.6)

SD, standard deviation; M_A_, model includes additive genetic effects; M_AL_, model includes additive genetic and litter effects; M_AP_, model includes additive genetic and pen effects; M_ALP_, model includes additive genetic, litter and pen effects

Based on group records for which phenotypes included litter and pen effects, variance components were estimated without bias by using the full model (M_ALP_). Models that ignored litter effects (M_AP_ and M_A_) overestimated the additive genetic variance but this overestimation was much less than the one when individual records were analyzed without litter effects, which indicates that only part of the litter effect moved to the additive genetic effect. The amount of overestimation of pen variance due to ignoring litter effects was similar to that based on individual records. Similar to analysis of individual records, the model that ignored pen effects (M_AL_) led to an unbiased estimate of the additive genetic variance, but overestimated the litter variance. Estimates of residual variances depended greatly on the model used for analysis. The model that ignored litter effects led to a slightly larger estimate of the residual variance and the model that ignored pen effects resulted in a very large estimate of the residual variance compared to the full model, which indicates that a large proportion of these effects merged into the residual effect. The largest estimate of the residual variance was obtained when using a model that ignored both pen and litter effects. These changes in estimated residual variances by different models based on group records are explained in the “Discussion” section.

For phenotypes without litter and pen effects, variance components that were estimated with the true model (M_A_) were unbiased regardless of whether individual or group records were used (Table 1). When the model included litter and pen effects, the resulting over-specification had a very small effect on estimates of variance components based on individual records, but had an impact on estimates based on group records. Using group records, the model that over-specified litter and pen effects resulted in small estimates of litter and pen variances, which slightly decreased the estimate of the additive genetic and residual variances.

The standard deviations of the estimated variance components from 100 replicates reflects their standard errors, which were very similar to the standard errors calculated with AI-REML (not shown). As expected, standard errors of estimates of variances based on group records were much larger than those based on individual records, which indicates a significant loss of information due to combining individual records into group records. When the model included pen effects (M_ALP_ and M_AP_), estimates of the residual variance had large standard errors, which highlights the difficulty to distinguish pen from residual effects based on group records.

### Prediction of BV using group records in different scenarios

As shown in Table 2, for simulated phenotypes with litter and pen effects, the models that ignored these effects greatly reduced the accuracy of EBV obtained from individual records, and those that ignored litter effects caused a serious inflation of EBV since the regression coefficient of BV on EBV was much less than 1. However, when analyzing group records, models that ignored litter and pen effects did not decrease the accuracy of EBV, while those that ignored litter effects led to a slight bias in EBV. The ratio of the accuracy of EBV obtained from group records to that from individual records was equal to 67.6%.

**Table 2:** Accuracy of EBV and bias (regression of true BV on EBV) [mean (SD) of 100 replicates] from different models with estimated variance components for the base scenario (L_2×3_)

Litter and pen effects simulated	Model	Group records		Individual records	
		Accuracy	Bias	Accuracy	Bias
Yes	M_A_	0.478 (0.038)	0.95 (0.107)	0.674 (0.019)	0.77 (0.028)
	M_AL_	0.478 (0.038)	1.01 (0.122)	0.693 (0.019)	1.00 (0.045)
	M_AP_	0.479 (0.038)	0.96 (0.108)	0.691 (0.018)	0.83 (0.028)
	M_ALP_	0.479 (0.038)	1.01 (0.121)	0.702 (0.019)	1.00 (0.042)
No	M_A_	0.550 (0.031)	1.01 (0.065)	0.714 (0.017)	1.00 (0.031)
	M_AL_	0.550 (0.031)	1.03 (0.069)	0.714 (0.017)	1.01 (0.031)
	M_AP_	0.550 (0.031)	1.02 (0.066)	0.714 (0.017)	1.00 (0.031)
	M_ALP_	0.550 (0.031)	1.03 (0.070)	0.714 (0.017)	1.01 (0.031)

EBV, estimated breeding value; SD, standard deviation; M_A_, model includes additive genetic effects; M_AL_, model includes additive genetic and litter effects; M_AP_, model includes additive genetic and pen effects; M_ALP_, model includes additive genetic, litter and pen effects

For phenotypes without litter and pen effects analyzed with a model that included these effects, the resulting over-specification did not decrease the accuracy of EBV regardless of whether individual or group records were used (Table 2). However, a slight deflation (reflected by the regression coefficient) of EBV was observed when using group records. This was in line with a small reduction of the estimate of the additive genetic variance due to over-specification. For phenotypes without litter and pen effects, the accuracy of EBV obtained from individual records increased slightly, while the accuracy of EBV obtained from group records increased considerably. The ratio of the accuracy of EBV obtained from group records to that from individual records was equal to 77.0%. These results indicate that the magnitudes of the litter and pen effects have a considerable influence on the accuracy of EBV when using group records.

Accuracies of EBV according to group structure are in Table 3. With a given group size, having more littermates in the same pen led to a higher accuracy of EBV when using group records, regardless of the existence of litter and pen effects. The accuracy of EBV decreased from 0.53 when all animals of a litter were in the same pen to 0.41 when animals were randomly distributed across pens for phenotypes with litter and pen effects, and from 0.60 to 0.48 for phenotypes without litter and pen effects. In contrast, when using individual records, the accuracy of EBV increased slightly as the number of littermates in each pen decreased for phenotypes with litter and pen effects, and hardly changed for phenotypes without litter and pen effects.

**Table 3:** Accuracy [mean (SD) of 100 replicates] of EBV for different structures of groups with sizes up to 12 (average 9.6)

Litter and pen effect simulated	Model	Placement of animals	Group records	Individual records
Yes	M_ALP_ with given true variances	A litter in 1 pen	0.529 (0.034)	0.696 (0.019)
		A litter in 2 pen	0.480 (0.038)	0.702 (0.019)
		Randomly in pens	0.407 (0.052)	0.706 (0.018)
No	M_A_ with given true variances	A litter in 1 pen	0.598 (0.027)	0.714 (0.017)
		A litter in 2 pen	0.550 (0.030)	0.714 (0.017)
		Randomly in pens	0.476 (0.038)	0.715 (0.017)

SD, standard deviation; EBV, estimated breeding value; M_A_, model includes additive genetic effects; M_ALP_, model includes additive genetic, litter and pen effects

**Fig. 1:**
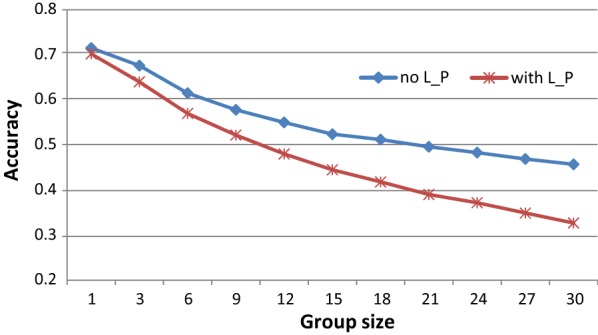
Accuracy (mean of 100 replicates) of EBV with different group sizes for phenotypes with or without litter and pen effects when three littermates together were allocated to a pen. The model used to predict BV was consistent with the model used to generate phenotypes

Figure 1 shows the accuracy of EBV obtained from group records when group sizes were changed from 3 to 30 (covering 1–10 sub-litters, respectively), as well as the accuracy obtained from individual records for the scenario of group sizes up to 12. In these cases, the model used to predict BV was consistent with the model that was used to generate phenotypes. Clearly, the accuracy of EBV tended to decrease with increasing group size. This decrease was more marked for phenotypes with litter and pen effects than for phenotypes without those effects. In the scenario of phenotypes without litter and pen effects, accuracies of EBV obtained from group records with group sizes up to 12 individuals (average 12*0.8 = 9.6) and up to 24 individuals (average 19.2) were equal to 77.0 and 68.4% of those predicted from individual records, respectively. In the scenario of phenotypes with litter and pen effects, these proportions were equal to 67.6 and 53.0%, respectively.

**Fig. 2:**
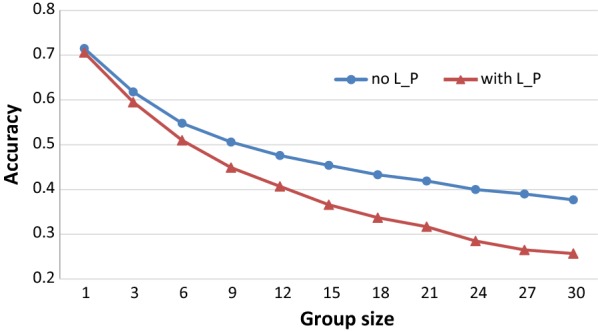
Accuracy (mean of 100 replicates) of EBV with different group sizes for phenotypes with or without litter and pen effects when animals were randomly allocated to pens. The model used to predict BV was consistent with the model used to generate phenotypes

Figure 2 shows that the accuracy of EBV tended to decrease with increasing group size in the scenario when animals were randomly distributed across pens. This trend was consistent with that in the scenario when a sub-litter of three littermates were in the same pen. Thus, for phenotypes without litter and pen effects, accuracies of EBV obtained from group records with group sizes up to 12 (average 9.6) and up to 24 (average 19.2) were equal to 66.6 and 55.9% of those estimated from individual records, respectively. In the scenario of phenotypes with litter and pen effects, these proportions were equal to 57.6 and 40.4%, respectively.

## Discussion

In this study, we extended the model for group records of Olson et al. [7] to the inclusion of multiple random factors such as litter and pen effects, as well as varying group sizes, and investigated the efficiency of using group records for genetic evaluation of feed intake in various scenarios. The extended model worked appropriately for analysis of the simulated data. The variance components estimated from group records were consistent with those estimated from individual records when the correct model was used, but had larger standard errors. Nevertheless, our results show that group records could be an important source of information for genetic evaluation of individual BV.

### Estimation of variance components

Our results show that variance components of a trait can be estimated from group records, but the estimates had larger standard errors than those obtained from individual records. Variance components estimated from group records were consistent with those estimated from individual records when the correct model was used, i.e., the model that included litter and pen effects if they existed. When litter and pen effects were present but ignored in the model of analysis, variances estimated from group records and from individual records were both biased but to different degrees. When litter effects were ignored, overestimation of the additive genetic variance was much larger when it was based on individual records than on group records. However, the change in the residual variances estimated with different models was much larger when they were based on group records compared to individual records. With the model that ignored litter or/and pen effect and used group records, the estimate of the residual variance depended on the number of littermates and the number of pen mates (i.e., group size in this study). In scenario L_2×3_, average $n_{g}$ is equal to 12*0.8 = 9.6, the average number of litters represented in a pen is equal to 4, and the average number of littermates is 3*0.8 = 2.4. When pen effects are ignored and assumed to move to the residual effects, $\sigma_{{{\mathbf{e}}_{{\mathbf{c}}}^{*} }}^{2} = n_{g}^{2} \sigma_{{\mathbf{c}}}^{2} + n_{g} \sigma_{{\mathbf{e}}}^{2} = n_{g} \left( {n_{g} \sigma_{{\mathbf{c}}}^{2} + \sigma_{{\mathbf{e}}}^{2} } \right) \approx 9.6^{*} \left( {9.6^{*} 40 + 200} \right) = 9.6^{*} 584$. Since $n_{g}$ is a diagonal element in ${\mathbf{R}}$, the estimate of the residual variance with this model is expected to be approximately 584 but a slightly lower estimate of 558 was observed (Table 1), because part of the pen variance actually moved to the estimate of the litter variance. Similarly, when litter effects are ignored and litter variance is assumed to move to residual variance i.e.

$$\begin{aligned} \sigma_{{{\mathbf{e}}_{{\mathbf{l}}}^{*} }}^{2} & = \mathop \sum \limits_{k = 1}^{{N_{l} }} n_{lk}^{2} \sigma_{{\mathbf{l}}}^{2} + n_{g} \sigma_{{\mathbf{e}}}^{2} \\ & = n_{g} \left( {\mathop \sum \limits_{k = 1}^{{N_{l} }} n_{lk}^{2} \sigma_{{\mathbf{l}}}^{2} /n_{g} + \sigma_{{\mathbf{e}}}^{2} } \right) \\ & \approx 9.6^{*} \left( {4^{*} 2.4^{2*} 40/9.6 + 200} \right) = 9.6*296, \\ \end{aligned}$$

thus, the estimate is expected to be 296. However, a much lower estimate of 226 was observed, because a large part of the litter variance moved to the additive genetic and pen variances. Finally, when both litter and pen effects are ignored and assumed to move to the residual effect, i.e.

$$\sigma_{{{\mathbf{e}}_{{{\mathbf{cl}}}}^{*} }}^{2} = n_{g} \left( {\mathop \sum \limits_{k = 1}^{{N_{l} }} n_{lk}^{2} \sigma_{{\mathbf{l}}}^{2} /n_{g} + n_{g} \sigma_{{\mathbf{c}}}^{2} + \sigma_{{\mathbf{e}}}^{2} } \right) \approx 9.6^{*} 680,$$

thus, the estimate from the model is expected to be 680. However, an estimate of 662 was observed, because part of the litter variance moved to the additive genetic variance.

Litter effects are usually included in the model to predict BV for growth traits [12–14]. In general, for feed efficiency in pig, BV is predicted based on individual records that are measured in a test station. Since in a test station only a relatively small proportion of the litters have more than one individual with records on feed intake, litter effects are often excluded from the prediction model [15, 16]. The current study showed that when there is a litter effect and more than one individual per litter is included in the dataset, a model that ignores litter effects results in overestimation of the additive genetic variance (Table 1), a reduced accuracy of EBV and an inflation of EBV (Table 2) when individual records are used. When group records are used, the model that ignores litter effects resulted in a slight overestimation of additive genetic variance and a slight inflation of EBV, but did not reduce accuracy of EBV in the simulated scenarios. Thus, our results suggest that litter effects should be included in the model for genetic evaluation of feed efficiency traits, even when using group records.

On the other hand, over-specification of the model by including factors that do not have an effect on the trait, also has an unfavorable impact on estimates of variance components, as shown in analyses in which litter and pen had no effect on the trait but the model included litter or/and pen effects. This could be because REML estimates must be larger than zero. Our study shows that the unfavorable effect of over-specification was larger when group records rather than individual records were used. This could be due to larger standard errors of estimates that are based on group records rather than individual records, and thus a wider range of REML estimates above zero. As observed in the current study, over-specification of litter and/or pen effects in the model led to underestimation of the additive genetic variance when group records were used, but had a very tiny impact when individual records were used.

Pen effects are often included in the prediction model for the genetic evaluation of growth and feed efficiency traits in pig [14–16]. However, based on group records, the estimate of the residual variance had a large standard error when the model included pen effects, and sometimes there was a problem with convergence for both estimation of variance components and prediction of BV, especially when the number of individuals varied slightly between pens. If the number of animals per pen is constant, pen effects cannot be separated from residual effects, which suggests that it can be difficult to distinguish between pen and residual effects.

An alternative to the full model for analysis of group records is a reduced model by combining the pen effects into residual effects, with appropriate weights on residuals. Thus, $\sigma_{{{\mathbf{e}}_{{\mathbf{c}}}^{*} }}^{2} = n_{g}^{2} \sigma_{{\mathbf{c}}}^{2} + n_{g} \sigma_{{\mathbf{e}}}^{2} = n_{g}^{2} b\sigma_{{\mathbf{e}}}^{2} + n_{g} \sigma_{{\mathbf{e}}}^{2} = n_{g} \left( {n_{g} b + 1} \right)\sigma_{{\mathbf{e}}}^{2}$, where $b = \sigma_{{\mathbf{c}}}^{2} /\sigma_{{\mathbf{e}}}^{2}$. Then, ${\text{V}}\left( {{\mathbf{e}}_{{\mathbf{c}}}^{*} } \right) = {\mathbf{R}}^{*} \sigma_{{\mathbf{e}}}^{2}$, where ${\text{R}}_{ii}^{*} = n_{gi} \left( {bn_{gi} + 1} \right)$. This reduced model is equivalent to the full model but greatly reduces computation demand and avoids the problem of convergence in the prediction of BV. When the dataset of group records is insufficient for accurate estimates of variance components, the ratio $b = \sigma_{{\mathbf{c}}}^{2} /\sigma_{{\mathbf{e}}}^{2}$ can be inferred from variance components estimated from individual records of feed efficiency, or other appropriate traits with individual records, such as daily gain.

### Efficiency of genetic evaluation based on group records

Given the same group size, having more littermates in the same group (pen) led to higher accuracy of EBV when group records were used. Our results confirmed previous reports that when relationships between individuals within a group were closer, the accuracy of EBV was higher when group records were used [7]. By theoretical derivation and data analysis, Peeters et al. [6] concluded that the accuracy of the estimate of additive genetic variance increased as the level of relationships between individuals within a group increased. Our study showed that even when using the true variance components, the accuracy of EBV increased as the degree of relationships between individuals in each group increased (Table 3). A possible reason could be that the proportion of additive genetic to phenotypic variance at the group level increases as the level of relationships between group mates increases. For example, assuming no genetic relationship between parents, based on Eqs. (4), (5), (6) and (7) and the parameters used in the simulation, if a group consists of four litters and each has three individuals, $\sigma_{{{\text{a}}^{*} }}^{2} = 120\left( {12 + 4\left( {6^{*} 0.5} \right)} \right) = 2880$, $\sigma_{{{\text{l}}^{*} }}^{2} = 40\left( {4^{*} 3^{2} } \right) = 1440$, $\sigma_{{{\text{c}}^{*} }}^{2} = 40\left( {12^{2} } \right) = 5760$, $\sigma_{{{\text{e}}^{*} }}^{2} = 200\left( {12} \right) = 2400$, and thus, the ratio of $\sigma_{{{\text{a}}^{*} }}^{2}$ to $\sigma_{{{\text{y}}^{*} }}^{2}$ is 0.23. However, this ratio is 0.31 when a group consists of two litters and each has six littermates.

In contrast, based on individual records, the accuracy of EBV decreased slightly as the number of littermates in the same pen increased when there were litter and pen effects. In a breeding program, the breeding goal usually includes many traits and most traits can be measured individually. Therefore, although more littermates in the same pen is favorable for genetic evaluation of feed efficiency based on group records, it is necessary to investigate whether this strategy is optimal for the overall breeding goal in a pig breeding program.

The presence of litter and pen effects caused a slight reduction in the accuracy of EBV when individual records were used, and a large reduction when group records were used. This could be due to a quadratic relationship between the variance of the cumulative litter effects and the number of littermates, and between the variance of the cumulative pen effects and the number of pen mates. Considering a scenario in which a pen includes four litters of three individuals, the ratio of $\sigma_{{{\text{a}}^{*} }}^{2}$ to $\sigma_{{{\text{y}}^{*} }}^{2}$ is 0.23 when there are litter and pen effects, but increases to 0.46 when there are no litter and pen effects, although heritability for individual records is the same in both cases.

We carried out an additional analysis to compare the accuracy of EBV based on group records and individual records for a trait with a low heritability, in the base scenario. In this analysis, data were simulated by setting $\sigma_{{\text{e}}}^{2}$ = 1000 for the scenario with litter and pen effects, and $\sigma_{{\text{e}}}^{2}$ = 1080 for the scenario without litter and pen effects, but without changing the other variance components. This led to a heritability of 0.10. Based on individual records, the accuracy of EBV was 0.57 and 0.59 for the scenarios with and without litter and pen effects, respectively. Based on group records, the accuracy of EBV was 0.41 and 0.44 for scenarios with and without litter and pen effects, respectively. The ratios of accuracies for group records to those for individual records were 71.4 and 75.6% in these two scenarios, respectively, which were similar to those for a trait with a heritability of 0.3. These results indicate that heritability has limited influence on the efficiency of using group records for predicting BV, in terms of the accuracy in relation to that when individual records are used. In other words, group records are valuable for predicting BV, regardless of the heritability of the trait.

In pig production systems, there are two popular structures of pens, i.e. (1) there is one feeder per pen (“single pen”), and (2) one feeder is shared by two pens (“double pen”). We used the example in which a single pen has a group size up to 12 (average 9.6) and a double pen has a group size up to 24 (average 19.6). Accuracies of EBV based on group records of the double pen were equal to 87.8% (phenotype without litter and pen effects) and 77.5% (phenotype with litter and pen effects) of the accuracies based on group records of the single pen, in the scenario with an average of 2.4 littermates in the same group. These proportions were equal to 84.0 and 70.0% in the scenario in which pigs were randomly allocated into pens. Therefore, to use group records efficiently, we recommend a pen structure with one feeder per pen.

The use of group records could also be valuable for predicting BV in other species. Biscarini et al. [5] used cage records to predict BV for body weight and egg production in laying hens. Cooper et al. [8] used pen records to predict BV for feed intake in beef cattle. Shirali et al. [4] used cage records to predict BV for feed intake in mink. The extended model in our study enables the handling of multiple random factors and varying group sizes, which will facilitate the use of group records for predicting individual BV in all these species.

Based on group records, full-sibs within group have the same EBV. When a multiple-trait model that includes traits with individual records (e.g., group records for feed intake and individual records for average daily gain) is used, full-sibs within a group can have different EBV for the group recorded trait. However, these differences in EBV between full-sibs within a group originate only from differences in the correlated traits between full-sibs. Genomic selection [17] has been successfully applied in pig breeding [18–20]. With genomic data, genotyped full-sibs within a group can have different EBV even when a single-trait model based on group records is used. In practical genetic evaluations, a good approach would be to predict BV for feed efficiency by a multiple-trait analysis using a single-step genomic model [21–23] and data that include individual (from a test station) and group records (from a farm) for feed efficiency together with individual records for correlated traits.

## Conclusions

Based on our findings, we conclude that the extended model for group records presented here can appropriately handle multiple fixed and random effects for estimation of variance components and prediction of BV using group records with varying group sizes. Estimates of variance components based on group records with unequal group sizes are consistent with those estimated from individual records when the correct model is used, although with larger standard errors. For traits such as feed intake in pigs, using group records for genetic evaluation of individual BV is feasible. For an efficient use of group records in genetic evaluation, group size should not be too large (e.g., a normal pen with its own feeder instead of two pens sharing a feeder for feed intake in pigs). In addition, a close genetic relationship between animals within a group is favorable for genetic evaluation based on group records, which should be taken into consideration when placing animals into groups (pens).

## Appendix

### An example to demonstrate the model and variance components

In this example, we use data from only two groups (Table 4), which is insufficient for estimating model parameters, but allows to exemplify the derivation of the model and variance components.

**Table 4:** Data for the example used to demonstrate the model and variance components

Id	Sire^a^	Dam^a^	Group (Pen)	Litter	Phenotype
P4	P1	–	–	–	–
P5	P1	–	–	–	–
1	P3	P2	1	1	11
2	P3	P2	1	1	12
3	P3	P2	1	1	13
4	P3	P4	1	2	14
5	P3	P4	1	2	15
6	P3	P4	2	2	16
7	P5	P6	2	3	18
8	P5	P6	2	3	17

^a^The sires and dams with unknown parents are not listed in the id column

The model for individual records is:

$$\begin{aligned} & {\mathbf{y}} = {\mathbf{Xb}} + {\mathbf{Z}}_{{\mathbf{l}}} {\mathbf{l}} + {\mathbf{Z}}_{{\mathbf{c}}} {\mathbf{c}} + {\mathbf{Z}}_{{\mathbf{a}}} {\mathbf{a}} + {\mathbf{e}}, \\ & \left[ {\begin{array}{*{20}l} {11} \hfill \\ {12} \hfill \\ {13} \hfill \\ {14} \hfill \\ {15} \hfill \\ {16} \hfill \\ {18} \hfill \\ {16} \hfill \\ \end{array} } \right] = \left[ {\begin{array}{*{20}l} 1 \hfill \\ 1 \hfill \\ 1 \hfill \\ 1 \hfill \\ 1 \hfill \\ 1 \hfill \\ 1 \hfill \\ 1 \hfill \\ \end{array} } \right]b + \left[ {\begin{array}{*{20}l} 1 \hfill & 0 \hfill & 0 \hfill \\ 1 \hfill & 0 \hfill & 0 \hfill \\ 1 \hfill & 0 \hfill & 0 \hfill \\ 0 \hfill & 1 \hfill & 0 \hfill \\ 0 \hfill & 1 \hfill & 0 \hfill \\ 0 \hfill & 1 \hfill & 0 \hfill \\ 0 \hfill & 0 \hfill & 1 \hfill \\ 0 \hfill & 0 \hfill & 1 \hfill \\ \end{array} } \right]\left[ {\begin{array}{*{20}c} {l_{1} } \\ {l_{2} } \\ {l_{3} } \\ \end{array} } \right] + \left[ {\begin{array}{*{20}l} 1 \hfill & 0 \hfill \\ 1 \hfill & 0 \hfill \\ 1 \hfill & 0 \hfill \\ 1 \hfill & 0 \hfill \\ 1 \hfill & 0 \hfill \\ 0 \hfill & 1 \hfill \\ 0 \hfill & 1 \hfill \\ 0 \hfill & 1 \hfill \\ \end{array} } \right]\left[ {\begin{array}{*{20}c} {c_{1} } \\ {c_{2} } \\ \end{array} } \right] + \left[ {\begin{array}{*{20}l} 0 \hfill & 0 \hfill & 0 \hfill & 0 \hfill & 0 \hfill & 0 \hfill & 1 \hfill & 0 \hfill & 0 \hfill & 0 \hfill & 0 \hfill & 0 \hfill & 0 \hfill & 0 \hfill \\ 0 \hfill & 0 \hfill & 0 \hfill & 0 \hfill & 0 \hfill & 0 \hfill & 0 \hfill & 1 \hfill & 0 \hfill & 0 \hfill & 0 \hfill & 0 \hfill & 0 \hfill & 0 \hfill \\ 0 \hfill & 0 \hfill & 0 \hfill & 0 \hfill & 0 \hfill & 0 \hfill & 0 \hfill & 0 \hfill & 1 \hfill & 0 \hfill & 0 \hfill & 0 \hfill & 0 \hfill & 0 \hfill \\ 0 \hfill & 0 \hfill & 0 \hfill & 0 \hfill & 0 \hfill & 0 \hfill & 0 \hfill & 0 \hfill & 0 \hfill & 1 \hfill & 0 \hfill & 0 \hfill & 0 \hfill & 0 \hfill \\ 0 \hfill & 0 \hfill & 0 \hfill & 0 \hfill & 0 \hfill & 0 \hfill & 0 \hfill & 0 \hfill & 0 \hfill & 0 \hfill & 1 \hfill & 0 \hfill & 0 \hfill & 0 \hfill \\ 0 \hfill & 0 \hfill & 0 \hfill & 0 \hfill & 0 \hfill & 0 \hfill & 0 \hfill & 0 \hfill & 0 \hfill & 0 \hfill & 0 \hfill & 1 \hfill & 0 \hfill & 0 \hfill \\ 0 \hfill & 0 \hfill & 0 \hfill & 0 \hfill & 0 \hfill & 0 \hfill & 0 \hfill & 0 \hfill & 0 \hfill & 0 \hfill & 0 \hfill & 0 \hfill & 1 \hfill & 0 \hfill \\ 0 \hfill & 0 \hfill & 0 \hfill & 0 \hfill & 0 \hfill & 0 \hfill & 0 \hfill & 0 \hfill & 0 \hfill & 0 \hfill & 0 \hfill & 0 \hfill & 0 \hfill & 1 \hfill \\ \end{array} } \right]\left[ {\begin{array}{*{20}l} {a_{p1} } \hfill \\ \ldots \hfill \\ {a_{p6} } \hfill \\ {a_{1} } \hfill \\ {a_{2} } \hfill \\ \ldots \hfill \\ {a_{7} } \hfill \\ {a_{8} } \hfill \\ \end{array} } \right] + \left[ {\begin{array}{*{20}l} {e_{1} } \hfill \\ {e_{2} } \hfill \\ {e_{3} } \hfill \\ {e_{4} } \hfill \\ {e_{5} } \hfill \\ {e_{6} } \hfill \\ {e_{7} } \hfill \\ {e_{8} } \hfill \\ \end{array} } \right]. \\ \end{aligned}$$

The covariance matrix of observations at the individual level is:

$$\begin{aligned} {\text{V}}\left( {\mathbf{y}} \right) & = {\mathbf{Z}}_{{\mathbf{l}}} {\mathbf{Z}}_{{\mathbf{l}}}^{{\prime }} \sigma_{{\mathbf{l}}}^{2} + {\mathbf{Z}}_{{\mathbf{c}}} {\mathbf{Z}}_{{\mathbf{c}}}^{{\prime }} \sigma_{{\mathbf{c}}}^{2} + {\mathbf{Z}}_{{\mathbf{a}}} {\mathbf{AZ}}_{{\mathbf{a}}}^{{\prime }} \sigma_{{\text{a}}}^{2} + {\mathbf{R}}\sigma_{{\mathbf{e}}}^{2} \\ & = \left[ {\begin{array}{*{20}c} {{\mathbf{J}}_{3 \times 3} } & 0 & 0 \\ 0 & {{\mathbf{J}}_{3 \times 3} } & 0 \\ 0 & 0 & {{\mathbf{J}}_{2 \times 2} } \\ \end{array} } \right]\sigma_{{\text{l}}}^{2} + \left[ {\begin{array}{*{20}c} {{\mathbf{J}}_{5 \times 5} } & 0 \\ 0 & {{\mathbf{J}}_{3 \times 3} } \\ \end{array} } \right]\sigma_{{\mathbf{c}}}^{2} + {\mathbf{A}}_{{\mathbf{s}}} \sigma_{{\mathbf{a}}}^{2} + {\mathbf{I}}_{8 \times 8} \sigma_{{\mathbf{e}}}^{2} , \\ \end{aligned}$$

where ${\mathbf{J}}$ is a matrix with elements being 1, ${\mathbf{A}}_{{\mathbf{s}}}$ is a subset of ${\mathbf{A}}$ as a relationship matrix for individuals with records, and ${\mathbf{I}}$ is an identity matrix.

The model for group records is: ${\mathbf{y}}^{*} = {\mathbf{X}}^{*} {\mathbf{b}} + {\mathbf{Z}}_{{\mathbf{l}}}^{*} {\mathbf{l}} + {\mathbf{Z}}_{{\mathbf{c}}}^{*} {\mathbf{c}} + {\mathbf{Z}}_{{\mathbf{a}}}^{*} {\mathbf{a}} + {\mathbf{e}}^{*}$, which can be constructed by:

$${\mathbf{Ty}} = {\mathbf{T}}\left( {{\mathbf{Xb}} + {\mathbf{Z}}_{{\mathbf{l}}} {\mathbf{l}} + {\mathbf{Z}}_{{\mathbf{c}}} {\mathbf{c}} + {\mathbf{Z}}_{{\mathbf{a}}} {\mathbf{a}} + {\mathbf{e}}} \right).$$

According to Table 4, the indicator matrix is:

$${\mathbf{T}} = \left[ {\begin{array}{*{20}l} 1 \hfill & 1 \hfill & 1 \hfill & 1 \hfill & 1 \hfill & 0 \hfill & 0 \hfill & 0 \hfill \\ 0 \hfill & 0 \hfill & 0 \hfill & 1 \hfill & 1 \hfill & 1 \hfill & 1 \hfill & 1 \hfill \\ \end{array} } \right].$$

Thus, the model for group records becomes:

$$\begin{aligned} \left[ {\begin{array}{*{20}c} {65} \\ {51} \\ \end{array} } \right] & = \left[ {\begin{array}{*{20}c} 5 \\ 3 \\ \end{array} } \right]b + \left[ {\begin{array}{*{20}l} 3 \hfill & 2 \hfill & 0 \hfill \\ 0 \hfill & 1 \hfill & 2 \hfill \\ \end{array} } \right]\left[ {\begin{array}{*{20}c} {l_{1} } \\ {l_{2} } \\ {l_{3} } \\ \end{array} } \right] + \left[ {\begin{array}{*{20}c} 5 & 0 \\ 0 & 3 \\ \end{array} } \right]\left[ {\begin{array}{*{20}c} {c_{1} } \\ {c_{2} } \\ \end{array} } \right] \\ & \quad + \left[ {\begin{array}{*{20}l} 0 \hfill & 0 \hfill & 0 \hfill & 0 \hfill & 0 \hfill & 0 \hfill & 1 \hfill & 1 \hfill & 1 \hfill & 1 \hfill & 1 \hfill & 0 \hfill & 0 \hfill & 0 \hfill \\ 0 \hfill & 0 \hfill & 0 \hfill & 0 \hfill & 0 \hfill & 0 \hfill & 0 \hfill & 0 \hfill & 0 \hfill & 0 \hfill & 0 \hfill & 1 \hfill & 1 \hfill & 1 \hfill \\ \end{array} } \right]\left[ {\begin{array}{*{20}c} {a_{p1} } \\ \ldots \\ {a_{p6} } \\ {a_{1} } \\ \ldots \\ {a_{8} } \\ \end{array} } \right] + \left[ {\begin{array}{*{20}c} {\mathop \sum \limits_{i = 1}^{5} e_{i} } \\ {\mathop \sum \limits_{i = 6}^{8} e_{i} } \\ \end{array} } \right]. \\ \end{aligned}$$

The covariance matrix of observations at the group level is:

$$\begin{aligned} {\text{V}}\left( {{\mathbf{y}}^{*} } \right) & = {\mathbf{Z}}_{{\mathbf{l}}}^{*} {\mathbf{Z}}_{{\mathbf{l}}}^{{*{\prime }}} \sigma_{{\mathbf{l}}}^{2} + {\mathbf{Z}}_{{\mathbf{c}}}^{*} {\mathbf{Z}}_{{\mathbf{c}}}^{{*{\prime }}} \sigma_{{\mathbf{c}}}^{2} + {\mathbf{Z}}_{{\mathbf{a}}}^{*} {\mathbf{AZ}}_{{\mathbf{a}}}^{{*{\prime }}} \sigma_{{\mathbf{a}}}^{2} + {\mathbf{R}}\sigma_{{\mathbf{e}}}^{2} , \\ & = \left[ {\begin{array}{*{20}c} {13} & 2 \\ 2 & 5 \\ \end{array} } \right]\sigma_{{\mathbf{l}}}^{2} + \left[ {\begin{array}{*{20}c} {25} & 0 \\ 0 & 9 \\ \end{array} } \right]\sigma_{{\mathbf{c}}}^{2} \\ & \quad + \left[ {\begin{array}{*{20}l} 0 \hfill & 0 \hfill & 0 \hfill & 0 \hfill & 0 \hfill & 0 \hfill & 1 \hfill & 1 \hfill & 1 \hfill & 1 \hfill & 1 \hfill & 0 \hfill & 0 \hfill & 0 \hfill \\ 0 \hfill & 0 \hfill & 0 \hfill & 0 \hfill & 0 \hfill & 0 \hfill & 0 \hfill & 0 \hfill & 0 \hfill & 0 \hfill & 0 \hfill & 1 \hfill & 1 \hfill & 1 \hfill \\ \end{array} } \right] {\mathbf{A}}\left[ {\begin{array}{*{20}l} 0 \hfill & 0 \hfill & 0 \hfill & 0 \hfill & 0 \hfill & 0 \hfill & 1 \hfill & 1 \hfill & 1 \hfill & 1 \hfill & 1 \hfill & 0 \hfill & 0 \hfill & 0 \hfill \\ 0 \hfill & 0 \hfill & 0 \hfill & 0 \hfill & 0 \hfill & 0 \hfill & 0 \hfill & 0 \hfill & 0 \hfill & 0 \hfill & 0 \hfill & 1 \hfill & 1 \hfill & 1 \hfill \\ \end{array} } \right]^{\varvec{'}} \sigma_{{\mathbf{a}}}^{2} \\ & \quad + \left[ {\begin{array}{*{20}c} 5 & 0 \\ 0 & 3 \\ \end{array} } \right]\sigma_{{\mathbf{e}}}^{2} . \\ \end{aligned}$$

Table 5 presents the solutions for the effects in the model using individual records and using group records, given variance components of $\sigma_{{\text{l}}}^{2}$ = 0.25, $\sigma_{{\text{c}}}^{2}$ = 0.25, $\sigma_{{\text{a}}}^{2}$ = 0.75 and $\sigma_{e}^{2}$ = 1.25.

**Table 5:** Solutions of the intercept (Int), pen effect (Pen), litter effect (Lit) and animal additive genetic effect (Anim) using individual records (Individual) and group records (Group) for the example

Item	Individual	Group	Item	Individual	Group
Int	14.81	14.94	Animp5	0.62	0.77
Pen1	− 0.48	− 0.79	Animp6	0.57	0.79
Pen2	0.48	0.79	Anim1	− 1.17	− 0.98
Lit1	− 0.52	− 0.47	Anim2	− 0.94	− 0.98
Lit2	0.14	− 0.05	Anim3	− 0.71	− 0.98
Lit3	0.38	0.52	Anim4	− 0.19	− 0.57
Animp1	0.39	0.35	Anim5	0.04	− 0.57
Animp2	− 0.78	− 0.71	Anim6	0.05	0.06
Animp3	− 0.57	− 0.79	Anim7	1.00	1.17
Animp4	0.36	0.12	Anim8	0.76	1.17
